# An Exploration of Emergency Healthcare Provision When Intimate Partner Abuse Is Identified

**DOI:** 10.1111/jocn.70252

**Published:** 2026-02-16

**Authors:** Shannon Dhollande, Silke Meyer, Diksha Sapkota, Karen‐Ann Clarke, Maria Atiénzar‐Prieto

**Affiliations:** ^1^ CQUniversity Brisbane Queensland Australia; ^2^ Griffith University Mount Gravatt Queensland Australia; ^3^ University of the Sunshine Coast Sippy Downs Queensland Australia

**Keywords:** domestic violence, emergency care, emergency treatment, intimate partner abuse, mental health

## Abstract

**Aim:**

This paper aims to synthesise the current, global evidence on addressing psychological concerns of women presenting with domestic and family violence within the ED and suggest avenues for future research.

**Design:**

This discursive paper draws on clinical experience and research of the authors and critical synthesis of current literature on management of victim‐survivors of DFV presenting with psychological symptoms in ED.

**Methods:**

Academic databases and grey literature were systematically searched to identify relevant sources, and findings were narratively synthesised.

**Results:**

DFV victim‐survivors often present with mental health symptoms in ED; however, many health professionals in EDs fail to correctly identify the underlying trauma and offer support to address DFV. The most reported barriers to DFV screening/identification include time constraints, privacy issues, and lack of education/training about DFV and its support mechanisms. As a result, only mental health symptoms are being treated, ignoring the broader psychosocial needs of DFV victim‐survivors. Use of trauma‐informed support models is recommended to address the mental and psychosocial needs of DFV victim‐survivors visiting the ED.

**Conclusions:**

DFV victim‐survivors visiting the ED are often treated for their mental health symptoms without addressing their underlying trauma and risk of future victimisation. To address the ongoing adverse impact of DFV, it is necessary to ensure holistic and continual support from ED professionals for victims.

**Implications for the Profession and Patient Care:**

The importance of not only education but the implementation of sustained education and training programs surrounding DFV identification, screening, and cannot be understood. DFV is a global problem whereby many victim‐survivors become healthcare patients. It would be poor decision making for clinicians to not prioritise appropriate responses to this societal problem within their clinical practice.

## Summary

- What does this paper contribute to the wider global clinical community?
  ○ Mental health symptoms are common reasons for emergency department visits by victim‐survivors of domestic and family violence.
  ○ Despite evidence advocating the use of trauma‐informed practices to address underlying risk and trauma associated with DFV, little is known about the extent to which ED healthcare meets the needs for psychosocial support of victim‐survivors of DFV.
  ○ Clinicians need further education on the holistic needs of patients presenting with DFV related issues. Furthermore, emergency departments need to adapt their perspectives and models of care to meet the needs of their patients as key stakeholders.

## 1 Introduction

Domestic and family violence (DFV) is a pressing public health issue, with enormous medical, social and economic costs at an individual and societal level (Peterson et al. 2018; Wisner et al. 1999; KPMG 2016). DFV is a pervasive national and international health and welfare issue that affects between 17% and 23% of women in Australia, and one in three women worldwide (Australian Institute of Health and Welfare 2019; World Health Organisation 2021a). It is a complex and multifaceted phenomenon perpetrated by a partner or former partner often as a pattern of behaviours, and can encompass physical, sexual, psychological, emotional, and financial abuse (Australian Institute of Health and Welfare 2019; World Health Organisation 2021b). While it is acknowledged that women can perpetrate DFV against their male partners, women are overwhelmingly identified as victim‐survivors, particularly of more serious injuries and intimate partner homicide (Campbell et al. 2007; Hamberger and Larsen 2015; Wangmann et al. 2020).

Despite the severe effects of DFV, not all victim‐survivors seek help. Research indicates that, when women seek assistance, they tend to rely more on informal sources of help than formal ones, such as health care professionals (Fanslow and Robinson 2010). Formal help‐seeking decisions and strategies have been more commonly identified among women who experience more frequent and severe abuse, as well as those who report having suffered injuries requiring medical attention (Fanslow and Robinson 2010; Sabina and Tindale 2008; Meyer 2010). Women who have experienced DFV may utilise a number of health care services (Dienemann et al. 2000; Feder et al. 2011). Research has shown that, compared to other support services, health care providers are more frequently contacted by DFV victim‐survivors (Bowker and Maurer 1987). In fact, health care services may be the first point of contact for some women experiencing abuse from a partner (Koistinen and Holma 2015). These services include emergency departments (ED), where 1.1%–8% of the visits have been found to be associated with DFV (Farchi et al. 2013; Parekh et al. 2012). In large regional hospitals, ED presentations may be commonly associated with DFV due to a lack of other community services (Baird et al. 2019). Further, research by Singhal and colleagues (Singhal et al. 2021) showed that among 10,935 DFV‐related ED visits, 61.5% (*n* = 6720) were associated with physical injuries, therefore, highlighting the high prevalence of DFV victim‐survivors presenting to the ED and the high prevalence rates of physical injuries among help‐seeking victim‐survivors. Considering this evidence, it is not surprising that EDs are the first point of contact with formal services for some victim‐survivors. Additionally, emergency care settings are commonly recognised as a safe space where DFV victim‐survivors are likely to disclose their abusive experiences (McGarry and Nairn 2015; Olive 2007). It is well‐established in the scholarly literature that a substantial number of women who attend EDs have experienced DFV at some point in their lives (Abbott et al. 1995; Boyle et al. 2006; Goldberg and Tomlanovich 1984) and the prevalence of DFV among women presenting to the ED is likely to be higher than in the general population (Olive 2007). In their literature review, Anglin and Sachs (Anglin and Sachs 2003) found that the 1‐year prevalence of DFV among female patients who presented to an ED ranged from 14.4% to 30%, while the lifetime prevalence of DFV ranged from 15% to 54%. Results from a systematic review by Sprague et al. suggest similar rates, with prevalence rates of DFV within the past year ranging from 11.5% to 36.0% among patients attending EDs, and lifetime prevalence rates between 16.1% and 51% (Sprague et al. 2014). When considering prevalence figures of DFV victim‐survivors' ED services usage, it is important to acknowledge that patients report difficulties in disclosing their experiences of abuse to ED professionals (Yam 2000), and women tend to underreport their experiences of DFV (Evans et al. 2016). Thus, figures regarding the prevalence rate of DFV‐related presentations in ED settings may be higher than the available data suggests.

## 2 Background

Whilst health care workers have been identified as key professionals in supporting the safety and wellbeing of women (Cronin and O'Connor 1993), many victim‐survivors are not effectively identified or provided with the necessary support (McGarry and Nairn 2015; Corbally 2001). Despite the high prevalence of victim‐survivors visiting EDs, Australian and international research evidence indicates that emergency professionals do not systematically screen for DFV (Sprague et al. 2014). Under‐identification of DFV cases in EDs is especially worrisome as research exploring intimate partner homicides shows that many women killed by a partner had been seen in an ED within the year prior to the intimate partner femicide (Brignone and Gomez 2017; Wadman and Muelleman 1999). These figures highlight the missed opportunities for ED professionals to provide support mechanisms, including adequate referrals, and safety plans for DFV victim‐survivors.

Women who experience DFV report significant short and long‐term consequences, including physical, psychological, social and financial negative impacts (Campbell et al. 2007; Golding 1999). Many victim‐survivors who have suffered severe injuries tend to seek care from EDs (Goldberg and Tomlanovich 1984), thus identification of DFV in emergency settings is commonly based on external injuries, such as contusions, lacerations or fractures (Farchi et al. 2013; Davidov et al. 2015). However, poor mental health has also been identified as a key presenting issue among DFV victim‐survivors. The most common adverse mental health outcomes associated with DFV identified in the literature are depression, anxiety, posttraumatic stress disorder (PTSD) (Coker et al. 2002; Lilly et al. 2015; Lövestad et al. 2017). For example, results from a study exploring symptoms and medical conditions of victim‐survivors who attended health services, including EDs, indicated that diagnoses of anxiety and depression were significantly associated with DFV (Eaton et al. 2016). In particular, help seeking behaviours for mental health problems were among the strongest predictors of current DFV victimisation (Eaton et al. 2016). Victim‐survivors of DFV also report higher levels of self‐harming behaviours, harmful substance use, and suicide (Beydoun et al. 2017; Dillon et al. 2013; García‐Moreno et al. 2013). Results from a retrospective study with a sample of 152 patients showed that 38.2% (*n* = 58) of patients consulting a psychiatric ED reported experiences of DFV (Leveque et al. 2023). Further, Australian research evidence indicates that mental health issues among DFV victim‐survivors presenting to the ED are common. Results from a retrospective study indicated that out of 56 cases of DFV, 48% (*n* = 27) of them had an associated mental health diagnosis (Ghafournia and Healey 2022). This highlights the prevalence rate of DFV among ED patients, including those presenting for physical injuries and mental health concerns. Severe and sustained victimisation is associated with greater severity of mental health symptoms (Dillon et al. 2013) Evidence suggests that, on average, victim‐survivors experience 35 episodes of abuse before seeking help from specialist services (Department of Health 2017). Therefore, it is likely that women attending ED present with severe negative mental health symptoms may be due to the repetitive nature of their victimisation. Research suggests that many DFV victim‐survivors have their emotional distress and related symptomology pathologised within their presentation in the ED and receive a concurrent mental health diagnosis (Beydoun et al. 2017). These diagnoses at times mask the underlying experiences of and support needs surrounding DFV. Adequate screening identification, risk assessment and intervention by health care providers is essential to meet victim‐survivors' support, safety and recovery needs; however, the absence of trauma‐informed practices in ED responses can hinder this process. The aim of this paper is to provide an overview of how health care professionals respond to the need for support of victim‐survivors who visit the ED, with a specific focus on the identification of mental health concerns and the need for psychosocial support.

## 3 Data Sources/Methods

An exploration of the literature was performed to examine and discuss the current state of knowledge in the focus area of this paper. Following the approach adopted by others (Zimmerman et al. 2016), a systematic review or meta‐analysis was not conducted as the nature of this review is exploratory, aiming to identify recurring themes in the area and suggest avenues for future research. A number of terms were identified to explore three dimensions with a search string for each one, including abusive behaviour (Dimension 1), setting (Dimension 2), mental health concerns (Dimension 3). The following four databases were searched to identify relevant studies: CINAHL, Web of Science, MEDLINE and Google Scholar. The final search string was adjusted where necessary taking into account functions unique to each database. The searches were conducted in April 2023 and they were limited to sources published in English. No date range or geographical region were applied. A manual search of the reference lists of the included literature was performed. Additionally, a manual search of research resources of key research bodies was also conducted, including ANROWS‐ Australia's National Research Organisation for Women's Safety Limited. These searches resulted in several publications and government reports. The search was to identify articles and documents that examined: responses by health care professionals to presentations of DFV victim‐survivors to the ED and how victim‐survivors' mental health complaints are dealt with. The aim of this paper was to identify the extent of existing research evidence in the area; thus, quality was not used as a criterion for exclusion. Documents such as journal articles, conference papers, reviews, theses, book chapters, articles in press, and reports (government and nongovernment) were included in the review. PowerPoint slides and community organisation websites or blogs were excluded. Data on participant characteristics (sex, age, ethnicity), study characteristics (country, year of publication, sample size, study design), and key findings were extracted, where applicable.

## 4 Overview of the Issues

After reviewing the studies, three dominant themes emerged following an inductive process. These include the DFV identification, DFV education and the medicalisation of victim‐survivors experiences. These are discussed further below.

### 4.1 DFV Identification

According to ED professionals, while physical signs of DFV are easier to recognise and manage, emotional and psychological indicators of DFV are seen as more complex and difficult to correctly identify (Hinsliff‐Smith and McGarry 2017).

### 4.2 DFV Education

Studies exploring health care professionals' education indicate that only around one‐third of clinicians had received training in DFV, which was often brief and lasted, on average, 1 to 3 h (Cohn et al. 2002; Fisher et al. 2020).

## 5 The Medicalisation of DFV Victim‐Survivors Experiences

Research indicates that health care professionals believe that they can recognise patients experiencing DFV based on their behavioural presentation, such as appearing distressed, tearful, nervous, hostile, fearful, excessively vigilant, evasive and/or reluctant to explain the cause of their injuries (Leppäkoski 2007). However, as Roelens and colleagues argue, most victim‐survivors may not exhibit overt indicators of abuse, but instead display a variety of complex symptoms (Roelens et al. 2006).

## 6 Discussion

Although the use of screening tools to identify and support women who experience DFV has been identified as effective, a lack of consistent utilisation of screening tools at an individual, institutional, and system level has been associated with negative health care experiences (Duchesne et al. 2022). Barriers to identifying and/or screening for DFV include time constraints, privacy issues, and emergency nurses' inappropriate assumptions or lack of education about DFV (Hinsliff‐Smith and McGarry 2017; Yonaka et al. 2007; Dowd et al. 2002), which may prevent the correct recognition of DFV as the underlying cause of ED presentations (Heinzer and Krimm 2002; Cohn et al. 2002). Additionally, ED settings are not commonly equipped with DFV support workers, specialist nurses or DFV advocates (Basu and Ratcliffe 2014), which can impede the identification and appropriate management of patients with DFV victimisation experiences.

Research also suggests that emergency health care providers have questioned the appropriateness of using ED resources for the purpose of DFV screening (Dowd et al. 2002). In their focus groups study with emergency nurses and physicians, Dowd et al. found that, although health care staff were sensitive to the problem of DFV, time constraints and heavy workloads were identified as major obstacles for DFV screening (Dowd et al. 2002). Perceptions concerning whether DFV cases are deemed emergencies might be influenced in part by organisational attitudes towards prioritising DFV within practical responses. Recent research shows that service areas, including healthcare services, are more likely to screen for and respond to DFV where DFV is promoted as ‘core business’ through organisational leadership (Meyer et al. 2023).

Further education surrounding the importance of DFV identification may be essential in addressing attitudinal barriers. Results from a study assessing the introduction of an intensive training programme for medical and nursing staff within the ED showed an increase in DFV case detection rates and in staff's confidence in detecting DFV (Basu and Ratcliffe 2014). However, evidence suggests that while one‐off instructional training in isolation may improve ED staff awareness, these results do not translate to clinical practice and improvements are not maintained in the long term (Ansari and Boyle 2017). While some professionals in ED services have access to regular DFV training, research has identified barriers that hinder the effectiveness of this education. For instance, Baird and colleagues interviewed managers from 16 areas across an Australian hospital, including EDs, and found that, although ED staff had access to frequent DFV training, this tended to be ad hoc due to time pressures and staff availability (Baird et al. 2019). Given the limitations of the DFV training received by health care professionals, it is not surprising that they report low self‐ratings on knowledge and confidence in screening, supporting, and referring patients with DFV experiences (Soh et al. 2021). Moreover, research shows that some mental health professionals working within the emergency appear to be unclear of their role in identifying patients' experiences of DFV (Trevillion et al. 2012).

The lack of specialised DFV training among ED professionals together with the greater importance placed on screening for other health problems, as well as time and resource constraints present in EDs, may result in victim‐survivors' normal responses to DFV being confused with psychiatric disorders (e.g., Generalised Anxiety Disorder or Major Depressive Disorder) and pathologised within ED settings. The so‐called ‘psychopathologisation’ of victim‐survivors has been a main criticism by early feminist antiviolence activists in the context of healthcare provisions (Dobash and Dobash 1979). In their critical analysis, Lavis and colleagues (Lavis et al. 2005) argue that the adoption of the medical model of care may not only be ineffective in terms of service responses to DFV victim‐survivors but could also diminish the political significance of DFV as a social problem. In health care settings, such as EDs, failure to recognise DFV as the underlying cause of patients' clinical presentation may result in diagnoses that “deny, dismiss, and/or minimise the underlying contribution of domestic violence to women's ill health” (Lavis et al. 2005). This, in turn, can lead to unmet recovery and support needs for victim‐survivors.

Some researchers have developed mental health screening tools to aid emergency professionals in identifying patients who are at risk and referring them to mental health services (Houry et al. 2007). However, according to DFV victim‐survivors and some health care professionals, the predominance of a medical and diagnostic treatment model in clinical assessment leads to professionals primarily focusing on mental health symptoms resulting from victimisation experiences (Trevillion et al. 2012). Indeed, results from a validation study including 1138 female patient participants conducted by Houry et al. demonstrated that their brief mental health screening tool was highly predictive for depressive and PTSD symptoms and moderately predictive for suicidal ideation in DFV victim‐survivors (Houry et al. 2007). Based on these results, the authors concluded that valeted mental health scales can assist emergency physicians in referring DFV victim‐survivors to mental health services (Houry et al. 2007), however, other support needs, such as those at the psychosocial level, were not considered.

Exclusive focus on medical aspects may prevent the examination of underlying interpersonal, social, and systemic causes of DFV, resulting in health care staff overlooking the trauma associated with the abuse (Trevillion et al. 2012; Arkins et al. 2016). Mental health practitioners have reported that the process of medicalisation or the transformation of social problems into medical ones in the health services creates a barrier to considering patients health and emotional wellbeing in a holistic manner (Gillespie et al. 2022). Thus, within this model, health professionals are required to respond to the clinical mental health needs of a patient before exploring the presence of DFV where it may be argued that if the DFV was addressed there may not be mental health concerns (Gillespie et al. 2022). In the context of emergency settings, victim‐survivors presenting to an ED with mental health symptoms may receive medication and/or referrals to mental health services without necessarily receiving psychosocial responses required to address the underlying risk and trauma associated with DFV. Additionally, a lack of referral pathways has been identified by professionals as a key barrier to providing adequate support to victim‐survivors (Trevillion et al. 2012).

In their study, Spangaro and colleagues tested the feasibility of a systematic approach to routine screening and response for DFV among women presenting to the ED (Spangaro et al. 2020). Results showed that out of 868 women screened on first presentation at the ED, 18% (*n* = 154) reported DFV, and less than half of women that were positively screened (*n* = 75, 48.7%) received on‐site psycho‐social response (Spangaro et al. 2020)., Among the factors contributing to low numbers of psycho‐social referrals within EDs, the authors identified nurses' understanding of referrals as optional rather than a routine process, an absence of current DFV victimisation, and a lack of available social work referral mechanisms in rural regions (Spangaro et al. 2020). Lastly, apart from an absence of DFV training or education (Sutton et al. 2021), mental health practitioners also report a lack of knowledge of DFV support mechanisms (Gillespie et al. 2022). Thus, victim‐survivors referred to mental health services that are ill‐equipped to provide specialised care may still face challenges in receiving adequate support for their psychosocial needs.

## 7 Limitations

This review has several limitations. First, while a comprehensive search strategy was used and efforts were made to include a broad range of studies, it is important to note that, unlike a systematic review, the present discursive paper does not claim to be exhaustive or even a formal review. Therefore, it is possible that studies from peer‐reviewed publications and non‐traditional sources were not identified.

## 8 Conclusion

While a significant number of women attending EDs have experienced DFV (Bazargan‐Hejazi et al. 2014), research suggests that failure to detect and record DFV cases in ED settings is not rare (Corbally 2001). Various barriers have been shown to hinder the successful identification and management of DFV cases in ED settings (Yonaka et al. 2007; Heinzer and Krimm 2002), and when cases are recognised, a holistic and DFV specialised care approach may not always be adopted. The impact of DFV includes several mental health symptoms, such as psychological distress, anxiety, depression, substance abuse, self‐harm and complex trauma (Beydoun et al. 2017; Dillon et al. 2013; García‐Moreno et al. 2013; Salter et al. 2020). Within the ED these typical responses to DFV victimisation experiences may be mistaken for psychiatric disorders, thus masking underlying trauma and risk of ongoing harm. As a result, the primary care response may be a medical one, aimed at improving women's mental health outcomes and minimising risk to self and others. Overlooking victim‐survivors' psychosocial support needs around DFV not only hinders women's recovery, including mental health recovery, but further places victim‐survivors at ongoing and at times lethal risk of harm (Domestic and Family Violence Death Review and Advisory Board 2021).

While health services' failure to adequately respond to DFV has been criticised (Taket et al. 2003), policy initiatives have highlighted the significant role health professionals play in identifying and responding to DFV (Rabin et al. 2009; Department of Health 2008). Trauma‐informed care approaches have been proposed as an alternative to traditional medical treatment as it focuses on the impact of past and ongoing traumatic experiences, such as DFV victimisation, on a person's physical, psychological, and behavioural health (Gillespie et al. 2022). In Australia, the Queensland Domestic and Family Violence Death Review and Advisory Board (Domestic and Family Violence Death Review and Advisory Board 2021) has highlighted the need for DFV‐informed practice in healthcare responses. While patients value the screening for non‐medical needs such as psychosocial needs in health care settings (De Marchis et al. 2019; Byhoff et al. 2019), research suggests that health care professionals continue to encounter obstacles when meeting the nonmedical but intersecting social needs of patients visiting EDs (Malecha et al. 2018). Although screening for DFV is performed, more often screening is for other social needs, such as homelessness or food insecurity in ED settings (Samuels‐Kalow et al. 2021). Little is known about the degree to which ED healthcare meets the needs for psychosocial support in addition to medical treatment for victim‐survivors of DFV. Failure to acknowledge the role of trauma associated with DFV victimisation in mental health assessments can result in misdiagnosis and inappropriate and unsuccessful interventions (Gillespie et al. 2022). Therefore, to develop support and recovery mechanisms that meet the needs of victim‐survivors, it is necessary to better understand current gaps and future opportunities in healthcare and psychosocial support for women who experience DFV.

Despite the growing body of evidence around the benefits of trauma‐informed practices to address underlying risk and trauma associated with DFV, there is still a need to further understand the extent to which ED healthcare meets the needs for psychosocial support in addition to medical treatment for victim‐survivors once a mental health referral has been initiated. To identify the gaps in healthcare and psychosocial support for DFV victim‐survivors, future research should examine the provision of healthcare in EDs including the assessment, treatment, and referrals made for women experiencing DFV and mental health symptoms to inform future strategies that support holistic, trauma‐informed models of care for victim‐survivors of DFV.

## Funding

This work was supported by the University of the Sunshine Coast.

## Conflicts of Interest

The authors declare no conflicts of interest.
